# COVID-19 risk perception among residents of seven sub-Saharan African countries: socio-demographic correlates and predicted probabilities

**DOI:** 10.11604/pamj.2021.39.227.28193

**Published:** 2021-08-05

**Authors:** Ejemai Eboreime, Ihoghosa Iyamu, Barinaadaa Afirima, Emeka Franklin Okechukwu, Gabriel Isaac Kibombwe, Tolulope Oladele, Taurayi Tafuma, Okiki-Olu Badejo, Everline Ashiono, Mulamuli Mpofu, Edward Adekola Oladele

**Affiliations:** 1 Department of Psychiatry, Faculty of Medicine and Dentistry, University of Alberta, Edmonton, Canada,; 2 Department of Planning, Research and Statistics, National Primary Health Care Development Agency (NPHCDA), Abuja, Nigeria,; 3 Pan African Research Consortium, Federal Capital Territory, Abuja, Nigeria,; 4 School of Population and Public Health (SPPH), University of British Columbia, Vancouver, Canada,; 5 Family Health International (FHI360), Dar es Salaam, Tanzania,; 6 Family Health International (FHI360), Lusaka, Zambia,; 7 Community Prevention and Care Services Department, National Agency for the Control of AIDS (NACA), Abuja, Nigeria,; 8 Department of Public Health, Institute of Tropical Medicine, Antwerp, Belgium,; 9 Department of Paediatrics and Child Health, Egerton University, Nakuru, Kenya,; 10 EpiSolution Public Health Services, Federal Capital Territory, Abuja, Nigeria

**Keywords:** COVID-19, risk perception, sub-Saharan Africa, risk communication, pandemic

## Abstract

**Introduction:**

as the COVID-19 pandemic rages on, sub-Saharan Africa remains at high risk given the poor adherence to pandemic control protocols. Misconceptions about the contagion may have given rise to adverse risk behaviours across population groups. This study evaluates risk perception among 2,244 residents of seven countries in sub-Saharan Africa (Botswana, Kenya, Malawi, Nigeria, Tanzania, Zambia and Zimbabwe) in relation to socio-demographic determinants.

**Methods:**

an online survey was conducted via social media platforms to a random sample of participants. Risk perception was evaluated across six domains: loss of income, food scarcity, having a relative infected, civil disorder, criminal attacks, or losing a friend or relative to COVID-19. A multivariable ordinal logistic regression was conducted to assess socio-demographic factors associated with the perceived risk of being affected by COVID-19.

**Results:**

595 (27%) respondents did not consider themselves to be at risk, while 33% perceived themselves to be at high risk of being affected by the pandemic with respect to the six domains evaluated. Hospital-based workers had the highest proportional odds (3.5; 95%CI: 2.3-5.6) high perceived risk. Teenage respondents had the highest predictive probability (54.6%; 95% CI: 36.6-72.7%) of perceiving themselves not to be at risk of being affected by COVID-19, while Zambia residents had the highest predictive probability (40.7%; 95% CI: 34.3-47.0%) for high-risk perception.

**Conclusion:**

this study reveals the need to increase awareness of risks among socio-demographic groups such as younger people and the unemployed. Targeted risk communication strategies will create better risk consciousness, as well as adherence to safety measures.

## Introduction

In December 2019, the coronavirus disease 2019 (COVID-19) was identified as a novel contagion in Wuhan, China. The outbreak rapidly spread across the world, necessitating the declaration of a public health emergency of international concern (PHEIC) under the International health regulations by the World Health Organization on January 30, 2020, and as a pandemic in March 2020 [1,2]. However, the African continent seems to have been far less affected when compared to other continents. Various postulations have emerged as to why this is the case. Some of these postulations include limited testing capacity, younger age population, cross-immunity with prevalent infectious diseases, health systems preparedness following learnings from previous epidemics like Ebola Virus Disease, among others [3-6]. Yet, no definitive evidence has emerged on what the “protective” factors may be, empirically. Concerns have been raised, however, that the lower morbidity and mortality currently experienced in Africa may be evolving, while the continent remains largely unprepared following a false sense of invulnerability [3,7-9]. Many sub-Saharan African countries have reported widespread violations of safety measures and protocols aimed at curtailing the pandemic. There have also been misconceptions that COVID-19 is a disease of the higher socioeconomic class, or that certain locally consumed foods and substances provide protection from the virus [7, 10-11]. Consequently, there have been calls for contextually adapted risk communication and control measures in Africa [3,9,10,12,13]. Empirical and theoretical evidence demonstrate relationships between risk communication (how people “hear” warnings about potential hazards), risk perception (how people believe that they may be affected by potential hazards), and protective behavior (how people respond to what they hear about potential hazards by adopting preparation and mitigation measures) [14,15]. The degree and nature of risk communication often relate to the complexity of such a risk and the imminence of potential risk as well as risk perception [16]. Consequently, to adequately inform adherence with behavioral change measures (which have been the mainstay of most COVID-19 responses), effective risk communication is pertinent. The effectiveness of such a communication strategy depends on how interventions are appropriately targeted. Thus, an understanding of risk perception and related factors can significantly contribute to improving policies and strategies aimed at mitigating the spread of COVID-19 in Africa. This study evaluates the risk perception among residents of seven countries in sub-Saharan Africa, and the predicted probabilities of various levels of risk perception with socio-demographic determinants

## Methods

**Study design and sampling:** this study was a cross-sectional study across seven sub-Saharan African countries: Botswana, Kenya, Malawi, Nigeria, Zambia, Tanzania and Zimbabwe. The study used a random sample of respondents from 7 countries cutting across West (1), East/Central (2), and Southern Africa (4). These countries were selected to give a geographic representation across the different sub-Saharan African blocs which typically differ in national culture and context. Sample size was calculated using OpenEpi software [17]. The minimum sample size for each country at 95% confidence level was determined to be 384 respondents. Thus for all seven countries, a minimum sample size of 2,688 was determined. The formula used by OpenEpi software for sample calculation is:

$$n=\left[DEFF*Np\left(1-p\right)\right]/\left[d^{2}/Z_{1-\frac{\alpha }{2}}^{2}*\left(N-1\right)+P*\left(1-P\right)\right]$$

The elements of this formula as relates to this study are described as follows: i) population size (for finite population correction factor or fpc); (N): population of each country; ii) hypothesized % frequency of outcome factor in the population (p): 50%+/-5; iii) confidence limits as % of 100(absolute +/-%)(d): 5%; iv) design effect (for cluster surveys-DEFF): 1.

**Data collection tools and procedure:** the survey was administered online, between the 17^th^ May and 15^th^ June 2020 via a structured questionnaire that assessed respondents' socio-demographic characteristics and individual perception of being affected by the COVID-19 pandemic using a 5-point Likert scale. Perceived risk was assessed with respect to the following domains: loss of income, food scarcity, having a relative infected, civil disorder, criminal attacks, or losing a friend or relative to COVID-19. The questionnaire was created with appropriate skip patterns, pretested, and was administered via Google forms (Alphabet Inc, California, USA) online. These forms were then distributed via email, Facebook, Twitter, Telegram and WhatsApp using the following approach: initial call; i) Twitter-short survey intro plus link shared via Twitter adverts targeting the countries, broadcasts using hashtags of trending topics in each country and push through influencers in these countries; ii) Facebook-short survey intro plus link shared as broadcasts targeting each country; iii) WhatsApp and Telegram - short survey intro plus link broadcasts to individuals and groups by survey team in each country; iv) Telegram-short survey intro plus link shared using the groups nearby feature of Telegram that allows you to locate and share info with groups in a geographic location; v) Email listservs-short survey intro plus link shared to email listservs. Reminders: i) Repeated reminders till sample size was complete for the country via same channels as above; ii) Maximum of two via individual reminders to those who started but did not complete the survey. Data collection was closed after 28 days of initial launch or when the sample size had been completed. Respondents were eligible for inclusion into the study if they were, resident in one of the 7 countries considered in our study, aged ≥15 years, and able to communicate in English. We excluded non-residents and/or respondents who inadvertently gained access to the survey despite not meeting these criteria.

**Variables:** variables of interest in this study include socio-demographic profiles of respondents (age, sex, residential settings, the highest level of education, country of residence, and employment status). The primary response variable of interest was perceived self-risk of being affected by the COVID-19, assessed on a scale of zero to five, with zero representing “no perceived risk” and five representing very high perceived risk. Responses were then recategorized during data analyses into no perceived risk (0), low (1-2), moderate (3), and high (4-5) perceived risk). Other response variables collected include “What is the single most important factor that made you rate your risk this way?” with five categorical responses, as well as perceived likelihood of experiencing potential risks due to COVID-19 (loss of income, food scarcity, having a relative infected, civil disorder, criminal attacks, loss of friend/relative) were assessed on five-point Likert scale (very low, low, about the same, high and very high perceived risk).

**Data analyses:** data analyses were conducted using STATA 14.1 and R (version 4.0.2). All variables were initially summarized in frequency tables. Thereafter a multivariable ordinal logistic regression was conducted to estimate associations between socio-demographic variables and the primary response variable (perceived risk of being affected by COVID-19). Given the objectives of our study, we included all socio-demographic variables collected into the regression models. Post-estimation predicted probability modelling of perceived risk was conducted for each socio-demographic variable while holding all other variables constant at their respective means.

**Ethical considerations:** the survey protocol was approved by the Health Research Development Committee (HRDC) of the Ministry of Health and Wellness, the local institutional review board of Botswana (REF Number HPDME 13/18/1). A brief information page was the first section of the online questionnaire, and the respondents were allowed to consent electronically before completing the survey. Participation was voluntary and those who consented were allowed to exit the survey at any time by simply closing the browser page.

## Results

**Socio-demographic characteristics of participants:** descriptive characteristics of the respondents are provided in Table 1. Overall, 2244 respondents completed the survey, 1225 (55%) of whom were male. The median age group was 37 years (IQR: 27-42). 1447 (64%) of respondents were urban dwellers, and 2057 (92%) were educated up to the tertiary level. Most respondents live in Kenya, Botswana, and Nigeria: 568 (25%), 544 (24%), and 519 (23%), respectively. Most respondents were employed at the time of the survey, with 557 (25%) working with non-governmental organizations (NGOs). Private sector and government workers each accounted for about 17% of responses.

**Table 1:** socio-demographic variables

Variable		Frequency (n=2244)	Percent
**Age category (years)**	<20	40	1.8
	20-24	265	11.8
	25-29	340	15.2
	30-34	425	18.9
	35-39	444	19.8
	40-44	369	16.4
	45-49	180	8.0
	50-54	86	3.8
	>54	95	4.2
**Sex**	Female	954	42.5
	Male	1221	54.4
	Not stated	69	3.1
**Residential settings**	Rural	344	15.3
	Urban	1438	64.1
	Peri-urban	462	20.6
**Highest level of education**	Primary	38	1.7
	Secondary	157	7.0
	Tertiary	2049	91.3
**Country of residence**	Botswana	544	24.2
	Kenya	568	25.3
	Malawi	194	8.6
	Nigeria	519	23.1
	Tanzania	68	3.0
	Zambia	201	9.0
	Zimbabwe	150	6.7
Employment status	Unemployed	175	7.8
	Student	326	14.5
	Self-employed	286	12.7
	Retired	44	2.0
	Private sector	372	16.6
	Non-governmental organization	552	24.6
	Hospital-Based	110	4.9
	Government	379	16.9

**COVID-19 risk perception among respondents:**Table 2 describes the perceived risk of being affected by COVID-19 pandemic. 597 (27%) respondents did not consider themselves to be at risk, while 733 (33%) believed that they were at high risk of being affected by the pandemic. Of those who perceived themselves to be at risk, the majority (49%) cited occupational factors (profession or work environment) as the most important reasons for perceived risk. Only 51 (2%) respondents believed their home environment to be the single most important reason for their perceived risk rating. The majority (53-58%) of respondents thought that they were at increased (high or very high) risk of experiencing income loss, criminal attacks, friend/ relative infected, or dying due to the pandemic. However, the majority of respondents (52% and 58%, respectively) did not think they were at increased risk of experiencing civil disorder or food scarcity following the pandemic.

**Table 2:** summary of risk perception assessment

	Variable	Frequency n=2244	Percent
How do you rate your risk of being exposed to coronavirus?	No perceived risk	595	26.5
	Low	381	17.0
	Moderate	538	24.0
	High	730	32.5
What is the single most important factor that made you rate your risk this way?	N/A	595	26.5
	Work environment	674	30.0
	Inability to practice recommended measures	237	10.6
	Profession	413	18.4
	Existing health condition	88	3.9
	Home environment	51	2.3
	Means of transportation	186	8.3
How likely do you think you would experience any of the following due to COVID-19?			
Loss of income	Very low	143	6.4
	Low	356	15.9
	About the same	460	20.5
	High	654	29.1
	Very high	631	28.1
Food scarcity	Very low	208	9.3
	Low	526	23.4
	About the same	556	24.8
	High	524	23.4
	Very high	430	19.2
Having a relative infected	Very low	88	3.9
	Low	329	14.7
	About the same	573	25.5
	High	684	30.5
	Very high	570	25.4
Civil disorder	Very low	140	6.2
	Low	441	19.7
	About the same	583	26.0
	High	670	29.9
	Very high	410	18.3
Criminal attacks-burglary, robbery, etc.	Very low	122	5.4
	Low	360	16
	About the same	460	20.5
	High	661	29.5
	Very high	641	28.6
Losing a friend or relative to COVID-19	Very low	124	5.5
	Low	361	16.1
	About the same	571	25.4
	High	648	28.9
	Very high	540	24.1

**Predictors of the perceived risk of COVID-19:** outputs from the ordinal logistics regression are presented in Table 3. The regression model revealed that the proportional odds for respondents under 20 years of age being in a higher risk perception category was 0.3 (95% CI: 0.1 - 0.7) times less than those aged above 54 years (reference category) when the other variables in the model are held constant. Other age categories showed no statistically significant relationship. The proportional odds for respondents with the highest education at the primary level being in a higher risk perception category four times more than those with tertiary education (95% CI: 1.8 - 8.9). Also, residents of Botswana and Nigeria had lower proportional odds (0.6 (95% CI: 0.4-0.8) and 0.7 (95% CI: 0.5-0.9) times, respectively) for being in a higher perceived risk category than residents of Zimbabwe, holding the other variables constant. Further, respondents who were unemployed, self-employed, students, or working in the private sector had statistically significant lower proportional odds (0.4 (95% CI: 0.3-0.6), 0.4 (95% CI: 0.3-0.5), 0.4 (95% CI: 0.3-0.6), and 0.6 (95% CI: 0.5-0.8) times, respectively) for perceiving themselves as being at higher risk when compared to those working with governments. Conversely, when compared to respondents who were workers with governments, hospital-based workers had significantly higher proportional odds (3.6; 95% CI: 2.3-5.6 times) of being in higher perceived risk categories, holding other variables constant.

**Table 3:** adjusted ordinal logistic regression estimates of risk perception with 95% CIs

	Variable	Unadjusted odds ratios (95% CI)	Adjusted odds ratios(95% CI)
**Risk perception**	Zero/low		4.427*
	Low/Moderate		1.873*
	Moderate/High		0.623
**Age**	<20	0.226 (0.112-9.454)	0.310 (0.130-0.741)*
	20-24	0.544 (0.356- 0.829)*	0.913 (0.509-1.636)
	25-29	0.799 (0.526-1.214)	1.320 (0.775-2.248)
	30-34	0.937(0.623- 1.411)	1.303 (0.775-2.180)
	35-39	0.833 (0.555-1.250)	1.180 (0.709-1.963)
	40-44	0.843 (0.559-1.269)	1.096 (0.658-1.825)
	45-49	0.911 (0.580-1.429)	1.198 (0.696-2.064)
	50-54	0.899 (0.527-1.533)	1.162 (0.627-2.152)
	>54	(reference)	(reference)
**Sex**	Male	1.082 (0.929-1.261)	1.070 (0.913-1.254)
	Female	(reference)	(reference)
**Residence category**	Rural	0.929 (0.715-1.208)	1.003 (0.760-1.324)
	Urban	0.991 (0.794-1.236)	0.915 (0.743-1.126)
	Peri-urban	(reference)	(reference)
**Highest education**	Primary	1.495 (0.762-2.934)	3.980 (1.772-8.942)*
	Secondary	0.521 (0.382-0.711)*	0.932 (0.653-1.332)
	Tertiary	(reference)	(reference)
**Country of residence**	Botswana	0.524 (0.375-0.731)*	0.558 (0.388-0.803)*
	Kenya	0.836 (0.609-1.148)	1.143 (0.799-1.635)
	Malawi	1.233 (0.849-1.790)	1.094 (0.743-1.611)
	Nigeria	0.780 (0.566-1.075)	0.665 (0.476-0.928)
	Tanzania	1.347 (0.818-2.215)	1.234 (0.737-2.067)
	Zambia	1.471 (1.011- 2.138)	1.370 (0.931-2.016)
	Zimbabwe	(reference)	(reference)
**Employment status**	Unemployed	0.455 (0.326-0.635)*	0.433 (0.299-0.627)*
	Student	0.404 (0.309-0.548)	0.403 (0.276-0.590)*
	Self-employed	0.411 (0.309-0.541)	0.404 (0.298-0.546)*
	Retired	1.141 (0.631-2.063)	1.141 (0.532-2.444)
	Private sector	0.639 (0.049-0.830)*	0.642 (0.488-0.845)*
	Non-Governmental Organization	1.040 (0.823-1.131)	0.909 (0.708-1.168)
	Hospital-based	3.473 (2.259-5.339)	3.553 (2.274-5.551)*
	Government	(reference)	(reference)

**Figure 1:**
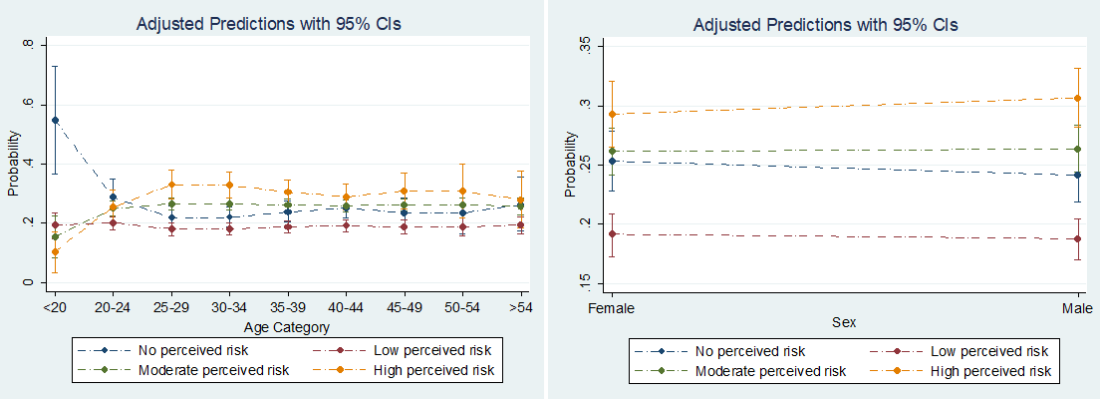
adjusted predictions for age and sex with 95% CIs

**Figure 2:**
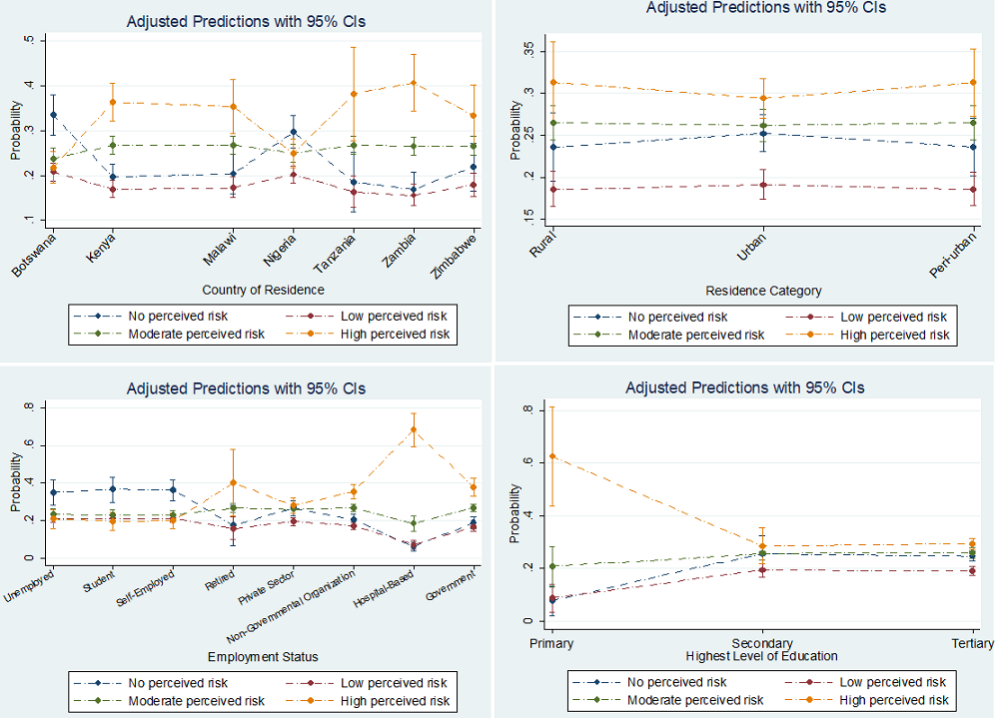
adjusted predictions for country, employment, residence, and highest education with 95% CIs

**Adjusted predicted probabilities of perceived risk categories with respect to each predictor**: Figure 1 and Figure 2 are error plots representing adjusted predicted probabilities of perceived risk for each predictor variable, holding other variables constant at their means. There were no statistically significant differences with respect to sex or residence category across all levels of risk perception. However, respondents who were less than 20 years of age had a 54.6% (95% CI: 36.6-72.7%) probability of perceiving themselves not to be at risk of being affected by COVID-19. The predictive probabilities of all other age groups were not statistically significant (Figure 1). Residents of all countries had relatively higher probabilities of perceiving themselves to be at high risk of being affected by COVID-19, with Zambia residents having the highest probability (40.7%; 95% CI: 34.3-47.0%). Residents of Botswana and Nigeria had a relatively higher probabilities of not perceiving themselves to be at risk, 33.5% (95% CI: 29.0-38.0%) and 29.7 (95% CI: 26.2-33.3%). The probability of falling into the “no perceived risk” category was relatively higher among the unemployed (34.9%, 95%CI: 28.1-41.8%), students (36.6%, 95%CI: 29.9-43.3%) and self-employed (36.6%, 95%CI: 31.1-42.1%) respondents. Respondents educated up to the primary level had the highest probability (62.7%, 95%CI: 44.1-81.4%) of being in the high perceived risk category when compared to other respondents (Figure 2).

## Discussion

This study generates and compares empirical evidence from seven African countries which could help improve social strategies to curb the ongoing pandemic. Like some other studies in similar African settings [8,18], this study demonstrates that most residents across all countries perceive themselves to be at some degree of risk of being affected by COVID-19. The extent of risk perception varied across countries (perhaps an indicator of contextual nuances in culture and response strategies), and with respect to other socio-demographic factors such as age, employment status and highest education. But unlike the observations of Iorfa *et al*. [8], our study findings indicate that risk perception did not vary statistically with sex or residential settings. Teenagers had a significantly high probability of not perceiving themselves to be at risk. This was not unexpected given the widespread knowledge that younger people are significantly less likely to experience severe forms of the disease than older people, even though young people are as much capable of transmitting the contagion [19-21]. Ordinarily, with this knowledge should come increased personal responsibility towards practicing precautionary measures [22]. This is, however, not always the case as many studies in Africa and across the world reveal that younger people are most likely to violate COVID-19 social interaction protocols [8,19,21]. Hospital workers had the highest probability of perceiving themselves to be at high risk of being affected by the pandemic. This is expected, given that health workers are in the frontline of efforts to control the pandemic, and constantly receive training and communications about the disease. On the other hand, students, self-employed and unemployed respondents had the highest probability of not perceiving themselves to be at risk of being affected. This may be partly because of the limited opportunities to interact physically due to school closures and movement restrictions. Further, younger people are more likely to fall into these work categories. Interestingly, our study also found that respondents whose highest education was at the primary level had a very high-risk perception probability, compared to respondents with secondary and tertiary education. In contrast to our sub-Saharan African study population, a study in Libya (North Africa) found college students to have a high-risk perception of being affected by COVID-19 [23].

Just 10% of respondents thought they would not be able to practice safety protocols. Key concerns that raised risk perception among the respondents were the potential for loss of income, relatives, and friends being infected or dying from COVID-19, and criminal attacks associated with the pandemic. Across the world, many have lost loved ones or income. However, perhaps counterintuitively, studies indicate that crime may not be on the increase following the pandemic [24]. One study in South Africa found a reduction in violent crimes following the pandemic [25]. While most respondents believed that work-related factors put them more at risk, there was a general feeling that the home environment portends minimal risk of being affected. However, some studies posit that overcrowding associated with living conditions in many sub-Saharan African contexts may pose a significant risk of infection at home [10]. Despite this risk, the low-risk perception could be leveraged by policymakers in the development of policies and strategies aimed at improving safe behavioral practices in the home environment. A key policy implication of this study is that it has made empirical evidence available to guide policymakers in designing and implementing targeted strategies, as against the more common “copy and paste” response model, which may not be very effective in this novel pandemic. Risk communication strategies could be targeted at age groups and occupational categories with low-risk perception. Policy makers could also consider the concerns of residents by planning for social and economic support during the pandemic, given that the increased economic burden tends to increase social interactions and intrinsic communal support systems which, though positive, could negatively impact control measures if not conducted with risk consciousness and precaution. This study has some limitations. Participants in this study were selected via a web-based strategy which intrinsically portends a selection bias with implications on the generalizability of findings. However, the safety precautions during the pandemic necessitated this approach. The findings can be viewed as an accurate estimate of risk perception among residents in these countries with internet access, given the considerably large sample size. Further, the information generated is useful to inform policies and strategies in these countries as well as other similar contexts. Future studies, using a sample more characteristic of the general population if feasible at the time, may complement our study findings.

## Conclusion

This study estimates the risk perception among residents in selected sub-Saharan African countries and highlights potential gaps that may be targeted by interventions to address the ongoing pandemic. Specifically, there is a need to increase awareness of risks among young people, as well as the unemployed. These categories of people, who may consider themselves to be less vulnerable to serious forms of the disease, are more at risk of the socioeconomic impact of the pandemic. Further, they are still susceptible to infection and unconscious spread of the contagion. Targeted enlightenment programs will go a long way to create better consciousness of risk, as well as the importance of pandemic control measures.

### What is known about this topic

- Sub-Saharan Africa remains at high risk of high morbidity and mortality due to COVID-19, given the poor adherence to pandemic control protocols;
- Misconceptions about the contagion may have given rise to adverse risk behaviours across population groups.

### What this study adds

- This study reveals the need to increase awareness of risks among socio-demographic groups such as younger people and the unemployed;
- Targeted risk communication strategies will create better risk consciousness, as well as adherence to safety measures.
